# Supplementary figures and images for: Reconstitution of EBV-directed T cell immunity by adoptive transfer of peptide-stimulated T cells in a patient after allogeneic stem cell transplantation for AITL
Source: PLoS Pathog. 2022 Apr 22;18(4):e1010206. doi: 10.1371/journal.ppat.1010206 (PMC9067708; doi:10.1371/journal.ppat.1010206)

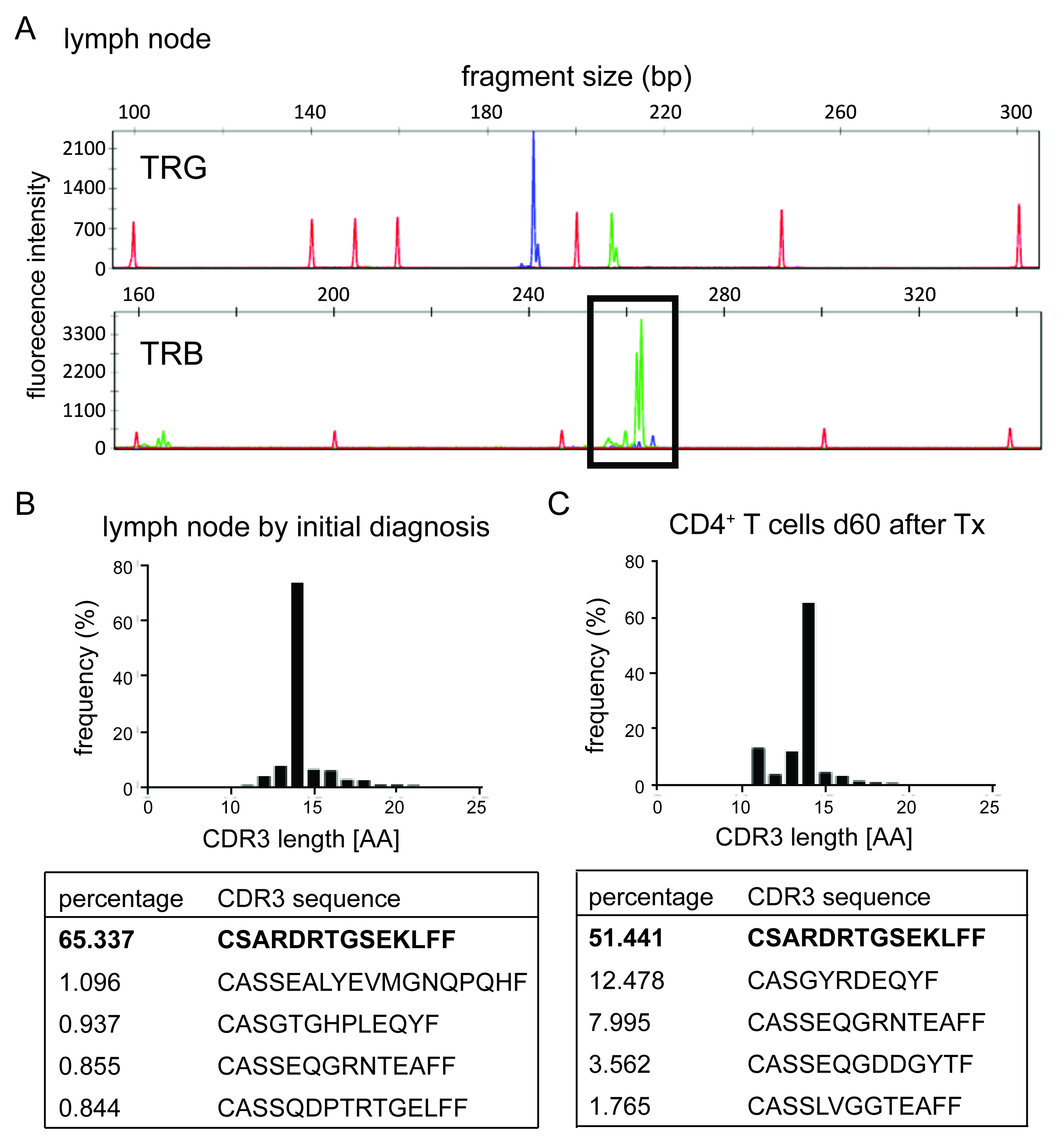

Supplement: S1 Fig — (A) GeneScan analysis of T cell receptor gamma (TRG) and beta (TRB) demonstrated clonal T cell populations in the lymph node biopsy (day 166 before transplant). (B) HTS of TCRβ rearrangements of the lymph node permit identification of the lymphoma-specific TCRβ sequence (in bold). (C) The lymphoma-specific TCRβ sequence could be identified again in the recipient on day 60 after transplantation in peripheral blood. (TIF) [file ppat.1010206.s001.tif]

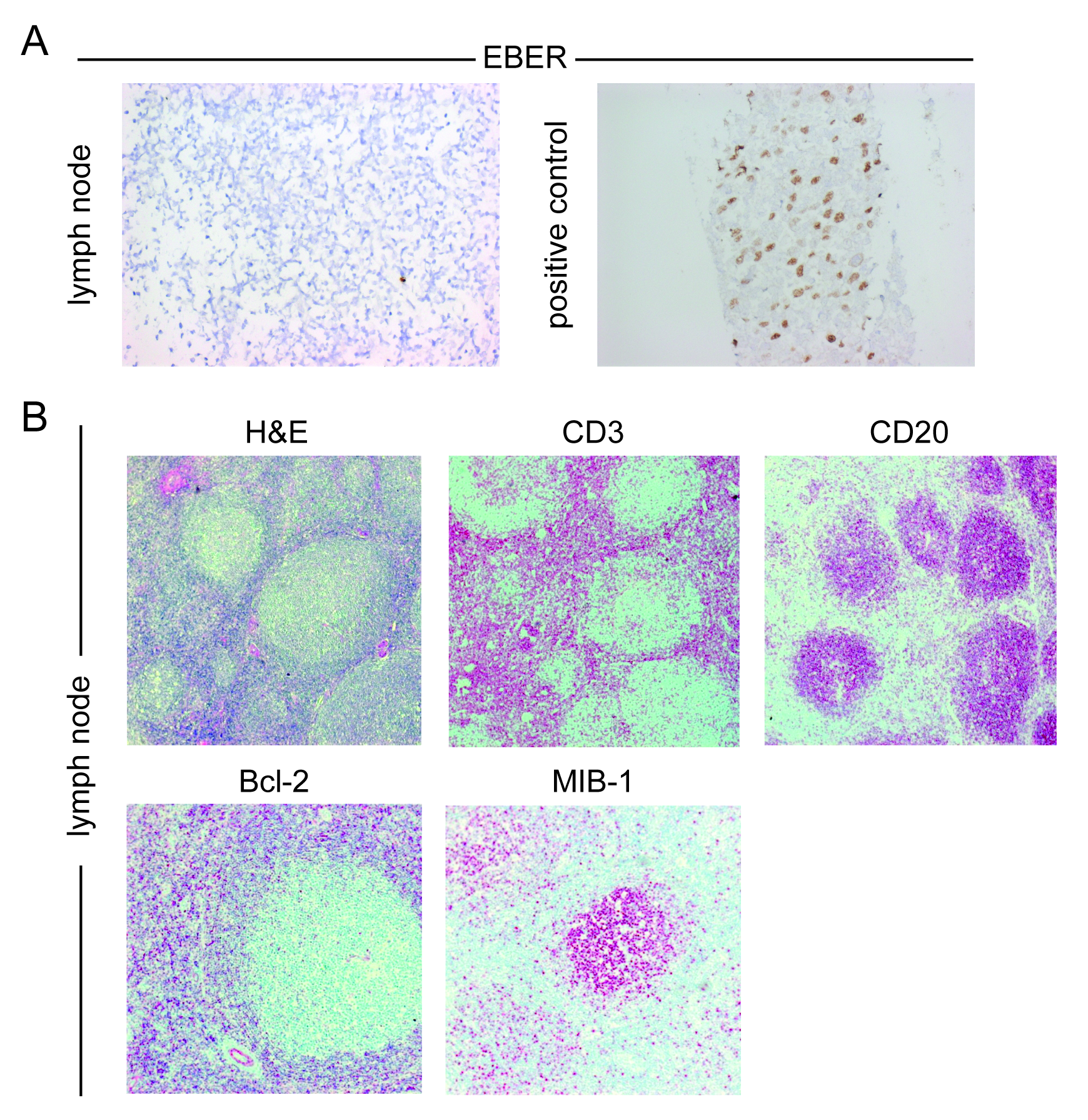

Supplement: S2 Fig — (A) EBER in situ hybridization (IsH) of the lymph node at diagnosis. An infectious mononucleosis sample was used as positive control. (B) Staining of the lymph node at diagnosis. H&E: Hematoxylin and eosin. (TIF) [file ppat.1010206.s002.tif]

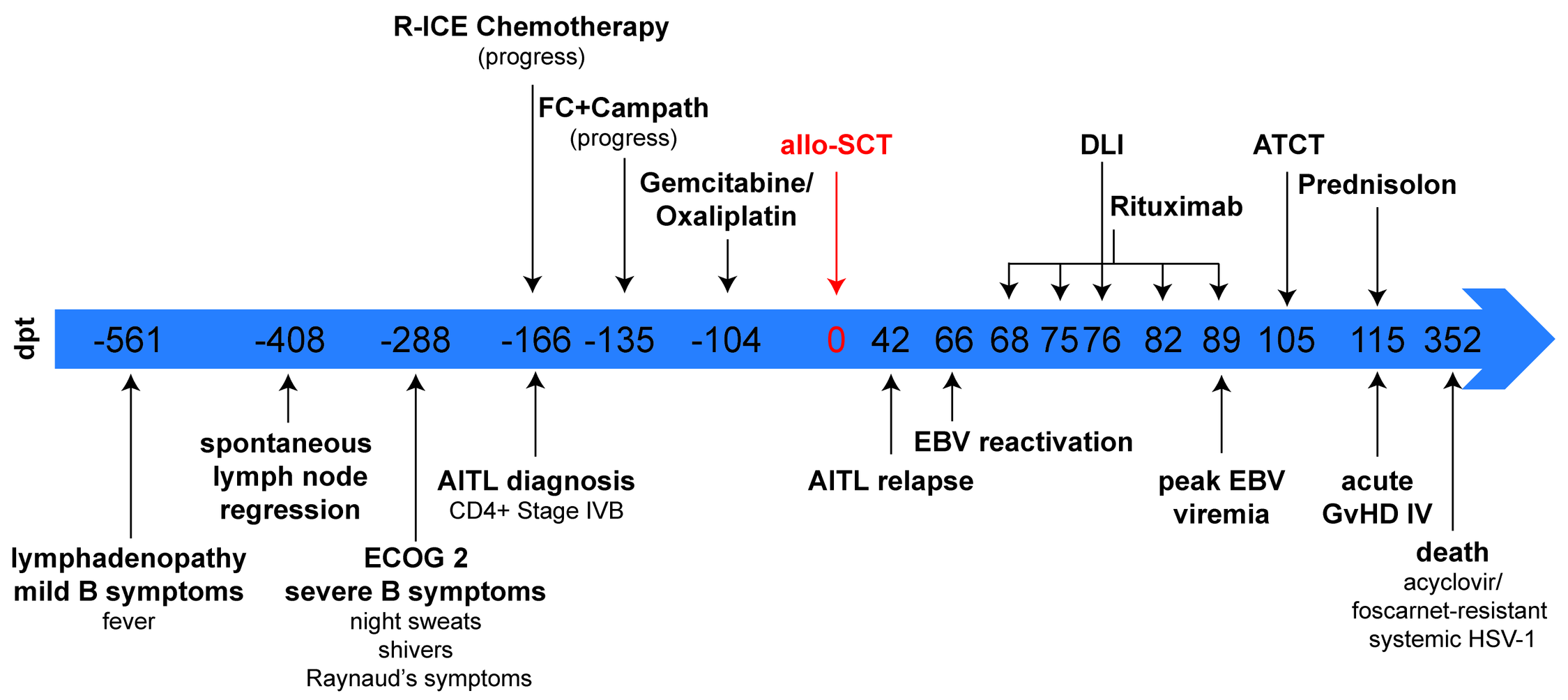

Supplement: S3 Fig — dpt: days post transplantation; ECOG: Eastern Cooperative Oncology Group; R-ICE: Rituximab, Ifosfamide, Carboplatin, and Etoposide Phosphate; GvHD: Graft-versus-Host Disease. (TIF) [file ppat.1010206.s003.tif]

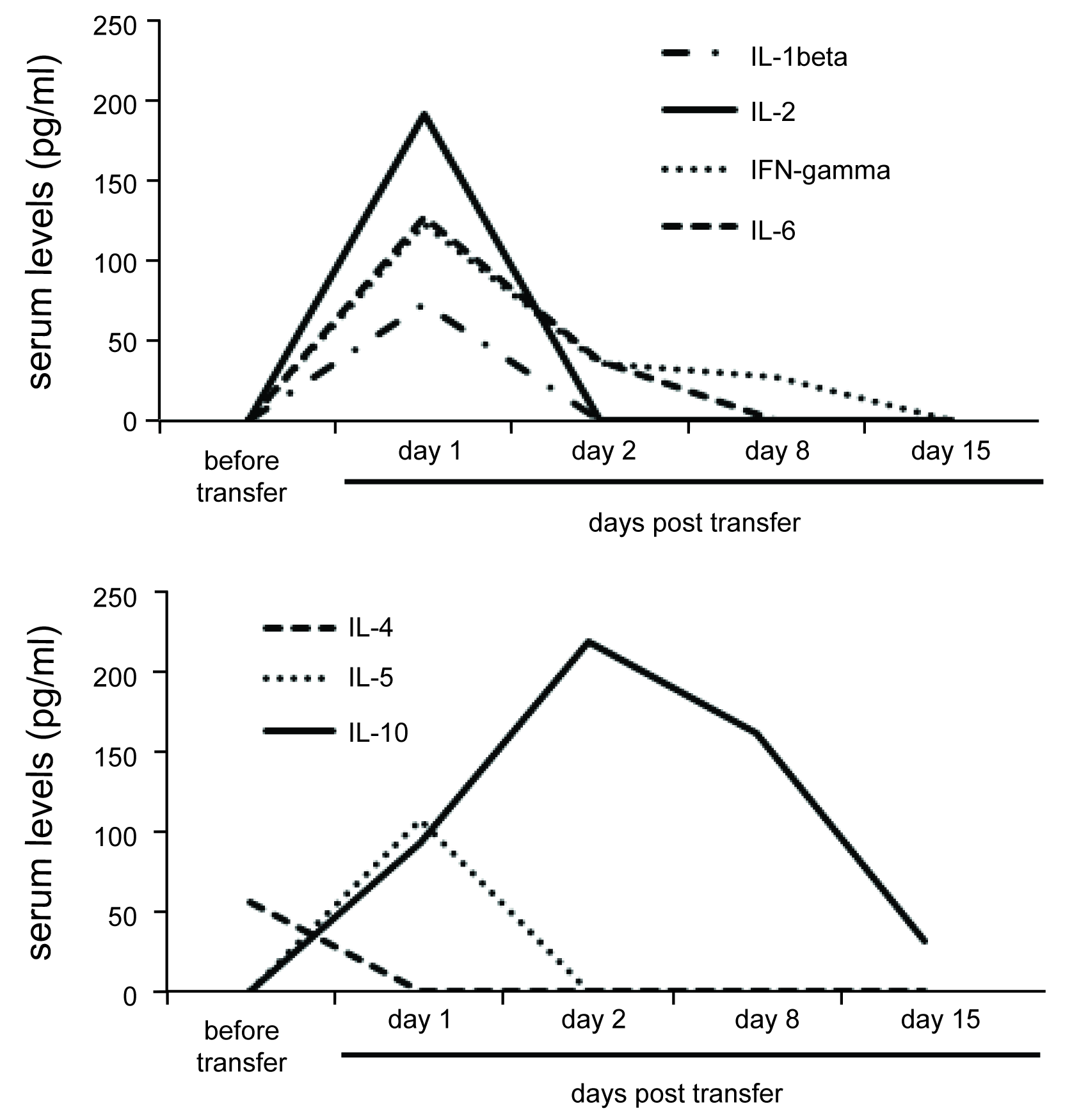

Supplement: S4 Fig — (TIF) [file ppat.1010206.s004.tif]

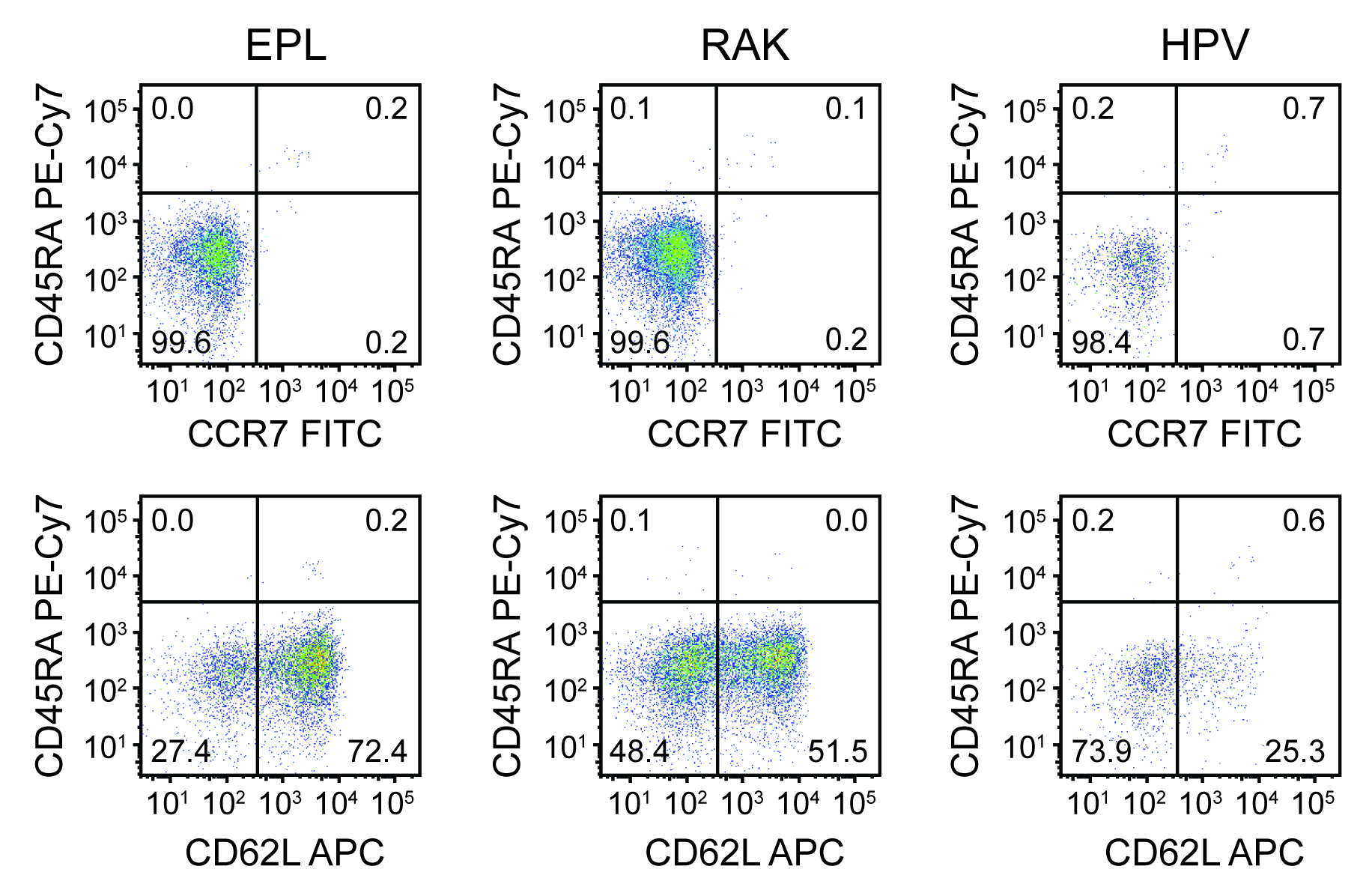

Supplement: S5 Fig — Multimer binding T cells were gated on CD8+ T cells. Plots were gated on multimer-binding T cells. Numbers indicate percentages. (TIF) [file ppat.1010206.s005.tif]

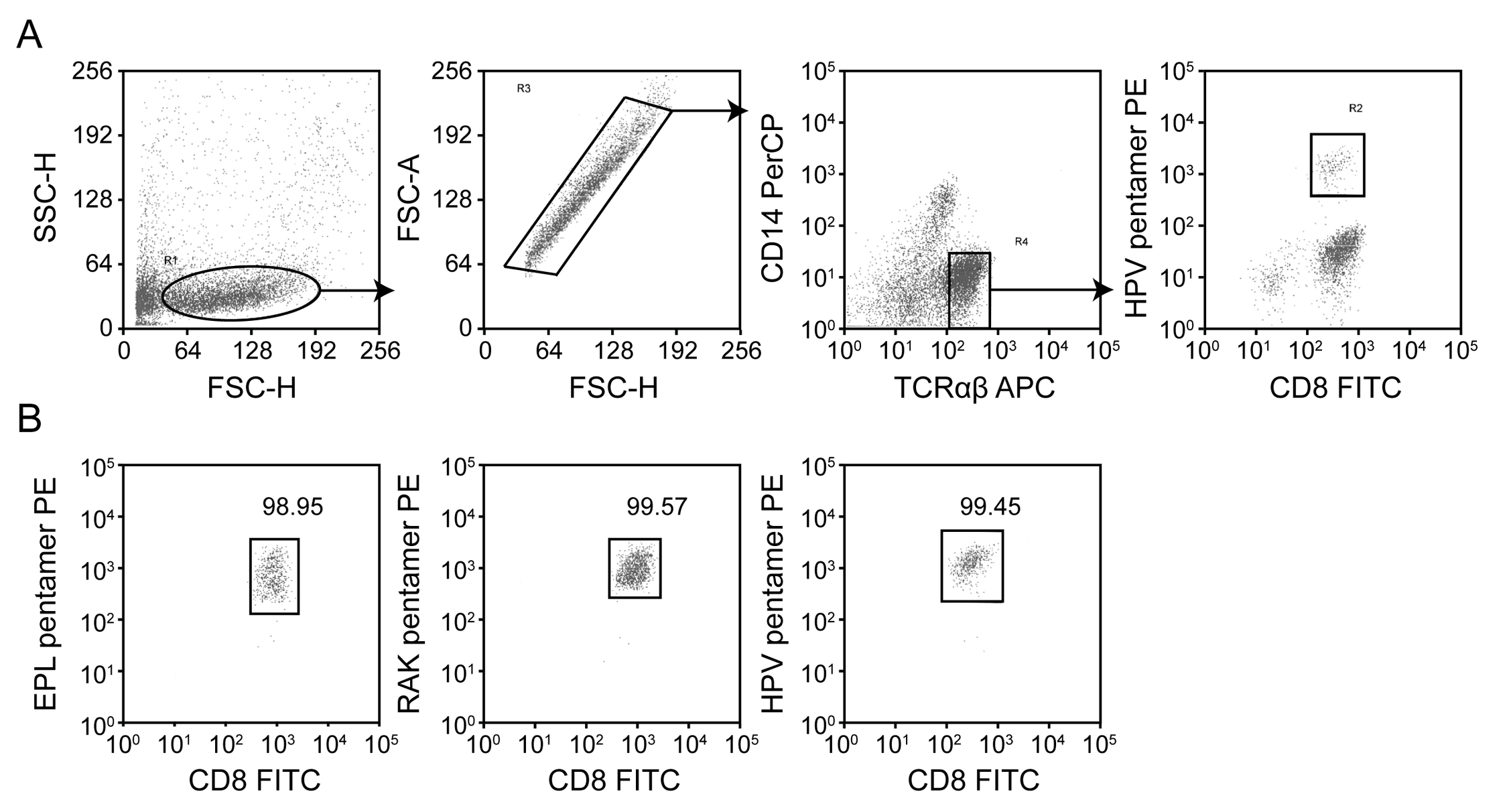

Supplement: S6 Fig — (A) Flow cytometric analysis gating for peptide-MHC multimer sorting. (B) Re-analysis of peptide-MHC multimer-sorted cells. Numbers indicate percentages. (TIF) [file ppat.1010206.s006.tif]

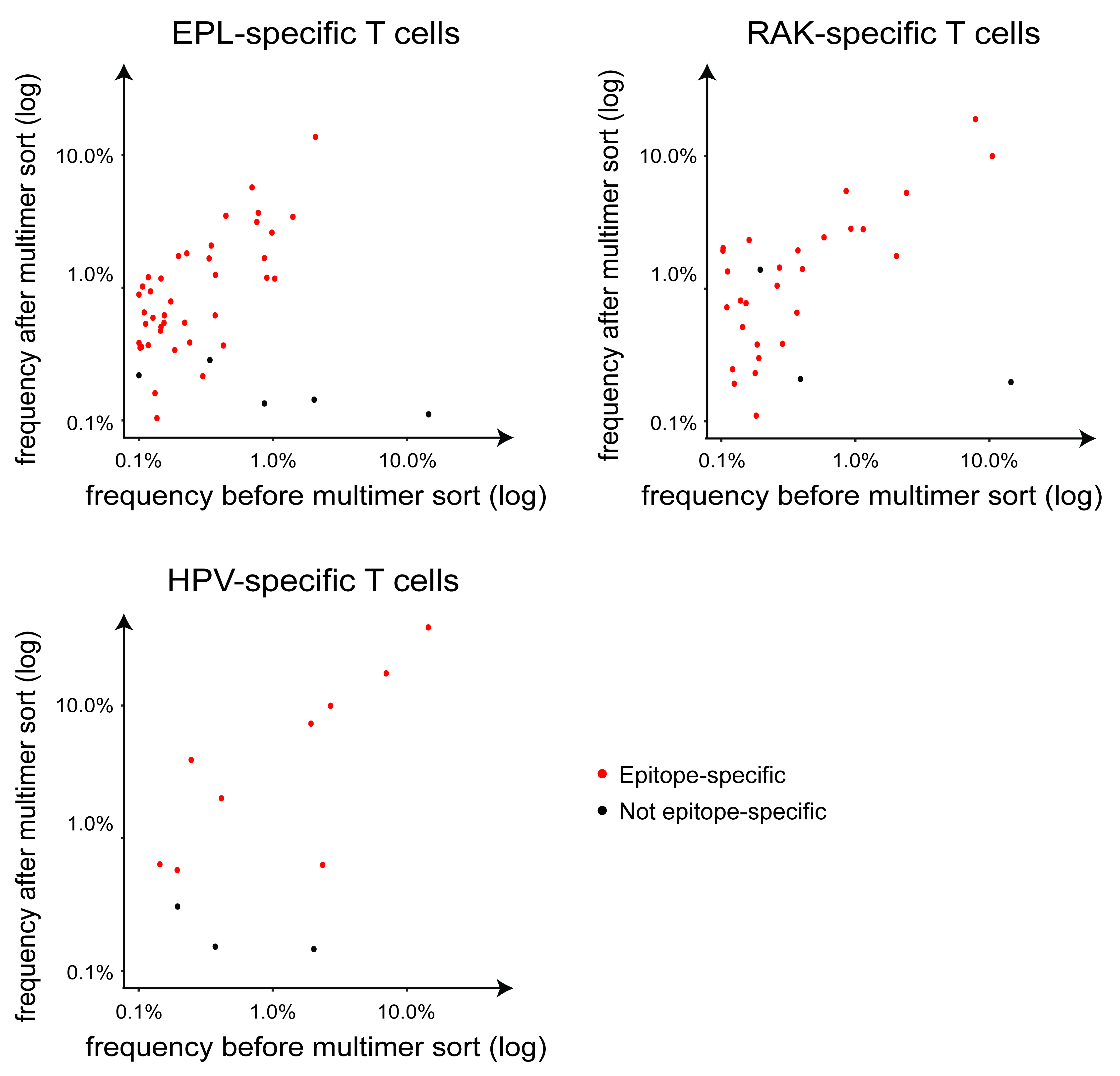

Supplement: S7 Fig — Scatter plots show frequency before and after multimer sort of the T cell product on day 9 of T cell clonotypes with a frequency above 0.1% in both populations. Each dot represents a single TCR clonotype. Red dots symbolize TCR clonotypes that pass a ternary exclusion criterion of at least ten times the multimer enrichment ratio for one multimer as compared with the other two and were, therefore, identified as epitope-specific. (TIF) [file ppat.1010206.s007.tif]

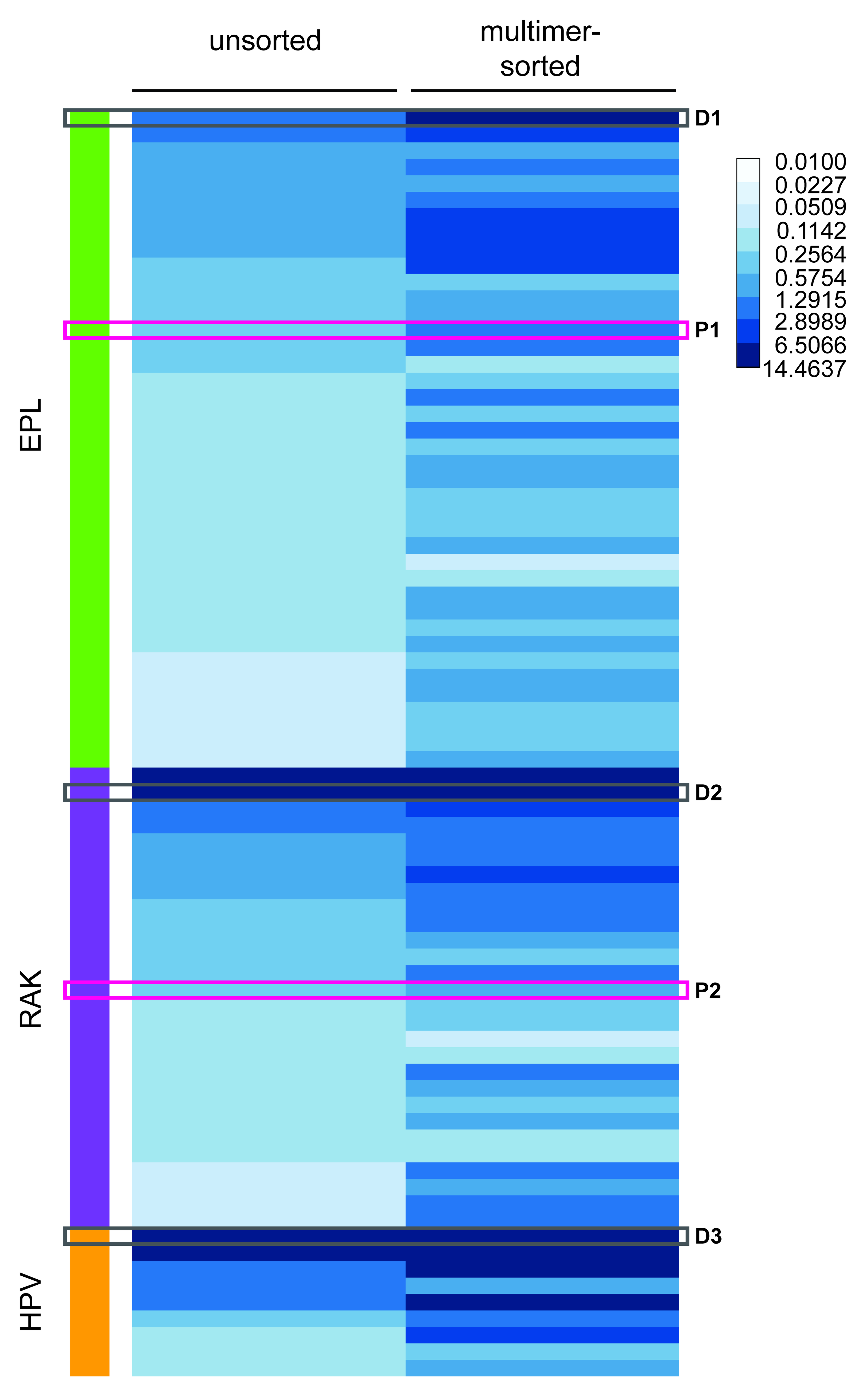

Supplement: S8 Fig — Frequencies of epitope-specific clonotypes (77 TCRs) is shown in unsorted CD8+ T cell product and MHC multimer-sorted sample. Clonotype frequency is displayed as a percentage from 0.01 (limit of detection) to 14.4637 by increasing colour depth. Each row represents one specific TCR rearrangement. Identified public TCR sequences (P1 and P2) published [28,30] are highlighted in grey boxes. The most dominant clones (D 1–3) within each specificity are highlighted in pink boxes. (TIF) [file ppat.1010206.s008.tif]

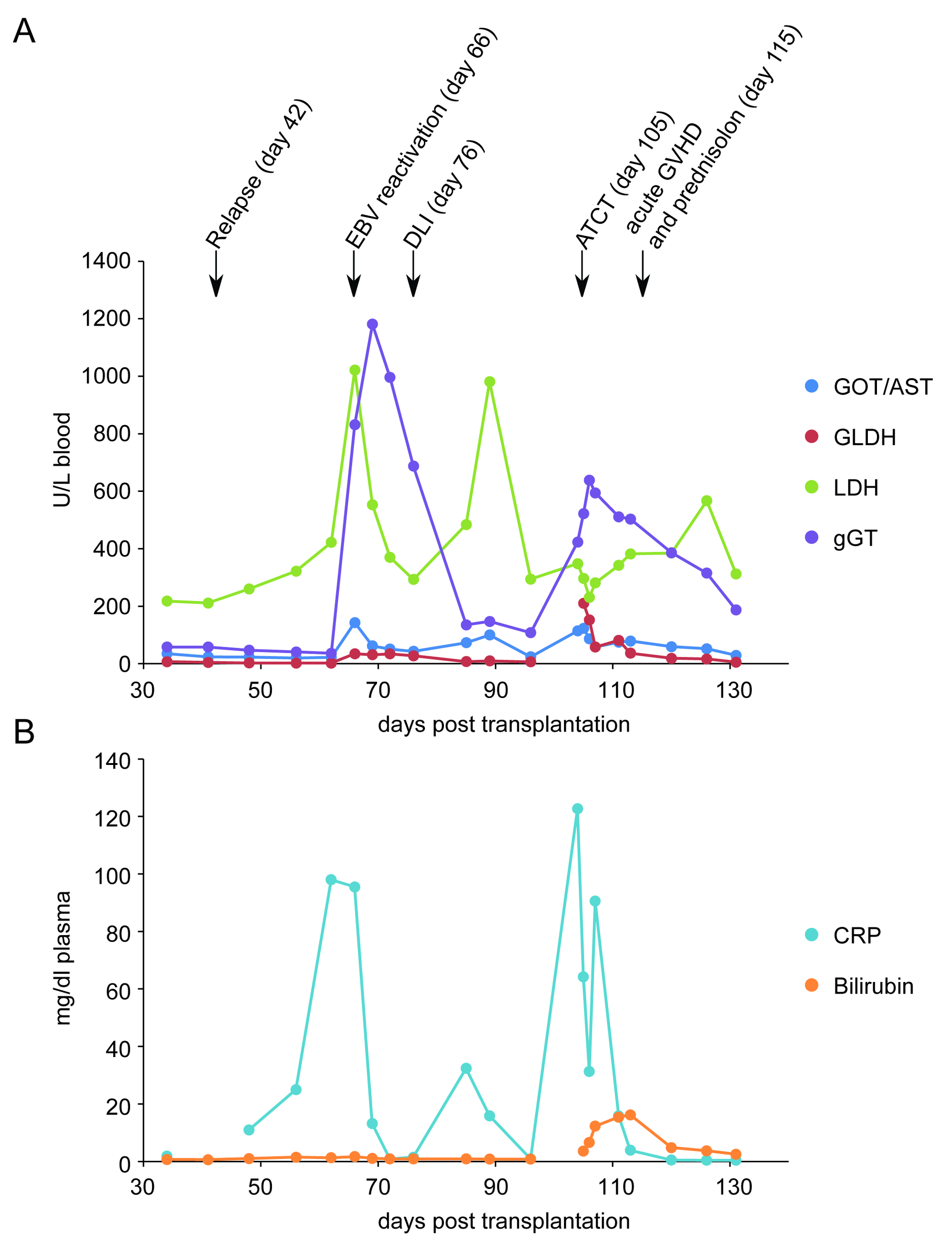

Supplement: S9 Fig — (A) Enzyme levels in blood and (B) biomarker levels in plasma were used to monitor GvHD progress. GOT: glutamic oxaloacetic transaminase, AST: aspartate transaminase, GLDH: glutamate dehydrogenase, LDH: Lactate dehydrogenase, gGT: gamma-glutamyltransferase, CRP: C-reactive protein. (TIF) [file ppat.1010206.s009.tif]
