# Supplementary material for: Reconstitution of EBV-directed T cell immunity by adoptive transfer of peptide-stimulated T cells in a patient after allogeneic stem cell transplantation for AITL
Source: PLoS Pathog. 2022 Apr 22;18(4):e1010206. doi: 10.1371/journal.ppat.1010206 (PMC9067708; doi:10.1371/journal.ppat.1010206)
Supplement: S1 Table — AITL: angioimmunoblastic T cell lymphoma; IgG: Immunoglobulin G; IgM: Immunoglobulin M; pos.: positive; neg.: negative; R-CHOP: Rituximab, Cyclophosphamide, Hydroxydaunomycin, Oncovin, and Prednisone; R-ICE: Rituximab, Ifosfamide, Carboplatin, and Etoposide; Fc: fragment crystallizable region; PD: progressive disease; SD: stable disease; allo-SCT: allogeneic stem cell transplantation; ATG: antithymocyte globulin; HLA: human leukocyte antigen; CMV: Cytomegalovirus; EBV: Epstein-Barr Virus; DLI: donor lymphocyte infusion; ATCT: adoptive T cell transfer; GvHD: Graft-versus-Host Disease; CsA: cyclosporin; HSV-1: Herpes-Simplex Virus-1. (PDF) [file ppat.1010206.s010.pdf]

| | |
|---|---|
| **age at diagnosis** | 55 |
| **diagnosis** | AITL, CD4+, Stage IVB |
| **EBV serology** | IgG pos., IgM neg. |
| **CMV serology** | IgG neg., IgM neg. |
| **MHC class I** | A*01:01, A*03:01, B*08:01, B*35:01, C*04:01, C*07:01 |
| **MHC class II** | DRB1*01:01, DRB1*03:01, DQB1*02:01, DQB1*05:01, DP not determined |

| **chemotherapy protocols** | **regimen** | **result/comment** |
|---|---|---|
| | 2 x Rituximab (375mg/sqm) | PD |
| | 2 x R-CHOP | PD |
| | 1 x R-ICE | PD |
| | 1 x Fc Campath | PD |
| | 6 x Gemcitabine/Oxaliplatinum | SD |

| | | |
|---|---|---|
| **allo-SCT**<br>**day 0** | conditioning regimen:<br>Thiotepa (10mg/kg)<br>Cyclophosphamide (60mg/kg)<br>Fludarabine (60mg/kg)<br>ATG (2.5mg/kg) | |
| **donor and graft** | HLA 10/10 match, male, CMV IgG neg., IgM neg., EBV IgG pos.<br>7.2 Mio./kg $CD34^+$ peripheral blood stem cells | relapse day 42, $CD4^+$ T cell counts rising in peripheral blood |
| **EBV reactivation**<br>**day 66** | Rituximab (375mg/sqm) weekly 4x<br>$1^{st}$ dose day 68 | max. viral load 140,000 genomic copies/ml on day 89 |
| **DLI**<br>**day 76** | 5.0 Mio./kg $CD3^+$ T cells day 76 | indication for relapse of AITL |
| **ATCT**<br>**day 105** | 1.0 Mio./kg $CD3^+$ peptide-stimulated T cells day 105 | control of EBV thereafter |
| **acute GvHD grade IV**<br>**day 115** | 2mg/kg Prednisolon, CsA | steroid responsive, improvement, and steroid taper |
| **outcome** | | death due to acyclovir/foscarnet - resistant systemic HSV-1 reactivation on day 352 |
