# Supplementary material for: Reconstitution of EBV-directed T cell immunity by adoptive transfer of peptide-stimulated T cells in a patient after allogeneic stem cell transplantation for AITL
Source: PLoS Pathog. 2022 Apr 22;18(4):e1010206. doi: 10.1371/journal.ppat.1010206 (PMC9067708; doi:10.1371/journal.ppat.1010206)
Supplement: S2 Table — TCRβ VJ ID: identification number for TCRβ variable-joining rearrangement, AA: amino acid. (PDF) [file ppat.1010206.s011.pdf]

EPL-specific T cells				
TCRβ VJ ID	CDR3 AA sequence	frequency in unsorted T cell product (%)	frequency in multimer-sorted sample (%)	fold enrichment
VJ-4201.65.1	CASRDRVGSEAFF	1.959	13.732	7
VJ-0405.69.1	CAISTGDSNQPQHF	1.339	3.425	3
VJ-2113.68.1	CSARDRGDTYEQYF	0.984	1.172	1
VJ-1907.65.1	CASRTGEVNEQFF	0.940	2.604	3
VJ-4005.69.1	CASSTGDVNQPQHF	0.862	1.189	1
VJ-4013.59.1	CASSYGPYEQYF	0.827	1.671	2
VJ-4206.69.1	CASSDSGIHNSPLHF	0.746	3.672	5
VJ-4011.66.1	CASRGGQGQETQYF	0.729	3.132	4
VJ-4207.71.1	CASSDSGTTFNEQFF	0.672	5.709	8
VJ-4201.71.1	CASSDTSALNTEAFF	0.432	3.487	8
VJ-4006.75.1	CASSYSSFRGGNSPLHF	0.427	0.367	1
VJ-3213.65.1	CASSPTQGFDEQYF	0.371	0.619	2
VJ-4202.69.1	CASSDTGQALYGYTF	0.362	1.249	3
VJ-2905.69.1	CASSTGDSNQPQHF	0.338	2.081	6
VJ-4202.72.1	CASSDTGQGPFYGYTF	0.325	1.664	5
VJ-1905.69.2	CASSTRDSNQPQHF	0.300	0.216	1
VJ-2405.72.1	CASSPDLYSNQPQHF	0.239	0.387	2
VJ-0305.71.1	CASGTFDSNQPQHF	0.223	1.817	8
VJ-0513.64.1	CAIAGTQFDEQYF	0.219	0.546	2
VJ-4201.65.2	CASSDSGMTEAFF	0.194	1.730	9
VJ-4012.68.1	CASGTGDPGANVLTF	0.185	0.339	2
VJ-4011.69.1	CASSLTTLDPETQYF	0.173	0.789	5
VJ-4013.80.1	CASSGVESSGTGLTYEQYF	0.155	0.620	4
VJ-2602.66.1	CASSLRGGLSGYTF	0.154	0.543	4
VJ-4013.59.2	CASSYGPYEQYF	0.146	0.507	3
VJ-2107.74.1	CSASGPGLYYNEQFF	0.145	0.475	3
VJ-1002.72.1	CASSLAAGTFLYGYTF	0.144	1.174	8
VJ-4013.59.3	CASSYGPYEQYF	0.136	0.104	1
VJ-1905.69.3	CASSTRDSNQPQHF	0.132	0.161	1
VJ-4202.72.2	CASSDSGQGGWDGYTF	0.127	0.594	5
VJ-2502.69.1	CASSLNPVPNYGYTF	0.121	0.939	8
VJ-4013.62.1	CASSDRVGVEQYF	0.117	0.369	3
VJ-2001.71.1	CASSETGVMNTEAFF	0.117	1.201	10
VJ-1909.83.1	CASSIDGIAGILASTDTQYF	0.112	0.535	5
VJ-4107.68.1	CASSYSPGIDEQFF	0.110	0.652	6
VJ-4202.69.2	CASSDTAQGLDGYTF	0.106	1.022	10
VJ-4206.69.2	CASSDSGTANSPLHF	0.105	0.358	3
VJ-5405.69.1	CASSLWGNNQPQHF	0.102	0.353	3
VJ-1902.69.1	CASSIPQGVPYGYTF	0.100	0.384	4
VJ-2006.69.1	CASHETGVGNSPLHF	0.100	0.888	9
