# Supplementary material for: Reconstitution of EBV-directed T cell immunity by adoptive transfer of peptide-stimulated T cells in a patient after allogeneic stem cell transplantation for AITL
Source: PLoS Pathog. 2022 Apr 22;18(4):e1010206. doi: 10.1371/journal.ppat.1010206 (PMC9067708; doi:10.1371/journal.ppat.1010206)
Supplement: S3 Table — TCRβ VJ ID: identification number for TCRβ variable-joining rearrangement, AA: amino acid. (PDF) [file ppat.1010206.s012.pdf]

RAK-specific T cells				
TCRβ VJ ID	CDR3 AA sequence	frequency in unsorted T cell product (%)	frequency in multimer-sorted sample (%)	fold enrichment
VJ-5613.71.1	CASSSQRQGRTYEQYF	10.491	9.985	1
VJ-3003.69.1	CASSTSRGAGNTIYF	7.862	18.947	2
VJ-3003.69.2	CASTSSRGGGNTIYF	2.407	5.294	2
VJ-3002.72.1	CASSQASYVQGDGYTF	2.028	1.761	1
VJ-2501.62.1	CASSSLNTEAFF	1.144	2.810	2
VJ-2713.65.1	CSAGQGEGYEQYF	0.925	2.834	3
VJ-3001.71.1	CASSQGLPLNTEAFF	0.853	5.449	6
VJ-2501.62.2	CASSSLNTEAFF	0.583	2.442	4
VJ-2501.62.3	CASSSLNTEAFF	0.403	1.409	3
VJ-3102.69.1	CASSQGTGFNYGYTF	0.373	1.946	5
VJ-5605.69.1	CASSPHPADQPQHF	0.366	0.660	2
VJ-3013.65.1	CASSPRQGESEQYF	0.286	0.385	1
VJ-3102.69.2	CASSQAEREDYGYTF	0.272	1.444	5
VJ-2501.62.4	CASSSLNTEAFF	0.261	1.053	4
VJ-3104.68.1	CASSQATGDNEKLFF	0.191	0.300	2
VJ-2506.60.1	CASSSLNSPLHF	0.185	0.380	2
VJ-0713.65.1	CASSLVAGSYEQYF	0.182	0.111	1
VJ-2713.62.1	CSGGQGPYEQYF	0.179	0.231	1
VJ-4807.74.1	CASSLIASGGYNEQFF	0.161	2.330	14
VJ-2501.62.5	CASSSLNTEAFF	0.153	0.779	5
VJ-2501.62.6	CASSSLNTEAFF	0.144	0.515	4
VJ-4801.77.1	CASSLVHSDYGLTEAFF	0.139	0.815	6
VJ-3113.74.1	CASSQEDSGLAVYEQYF	0.125	0.192	2
VJ-5606.81.1	CASSFSWSDQSSYNSPLHF	0.121	0.247	2
VJ-1312.77.1	CASSQVFWRGSGANVLTF	0.111	1.350	12
VJ-4009.65.1	CASTTTASTDTQYF	0.110	0.725	7
VJ-1307.74.1	CASSQGVTDYWNEQFF	0.103	2.025	20
VJ-3108.72.1	CASSPVTGNTGELFF	0.103	1.934	19
