# Supplementary material for: Reconstitution of EBV-directed T cell immunity by adoptive transfer of peptide-stimulated T cells in a patient after allogeneic stem cell transplantation for AITL
Source: PLoS Pathog. 2022 Apr 22;18(4):e1010206. doi: 10.1371/journal.ppat.1010206 (PMC9067708; doi:10.1371/journal.ppat.1010206)
Supplement: S4 Table — TCRβ VJ ID: identification number for TCRβ variable-joining rearrangement, AA: amino acid. (PDF) [file ppat.1010206.s013.pdf]

HPV-specific T cells				
**TCRβ VJ ID**	**CDR3 AA sequence**	**frequency in unsorted T cell product (%)**	**frequency in multimer-sorted sample (%)**	**fold enrichment**
VJ-4001.53.1	CASGTEAFF	14.464	38.810	3
VJ-4001.53.2	CASGTEAFF	7.020	17.472	2
VJ-4001.53.3	CASGTEAFF	2.700	9.966	4
VJ-4013.50.1	CASGNEQYF	2.359	0.630	0
VJ-4001.53.4	CASGSEAFF	1.929	7.303	4
VJ-4007.53.1	CASGNEQFF	0.414	1.998	5
VJ-1302.63.1	CASRPTGFDGYTF	0.247	3.890	16
VJ-4001.56.1	CASTWDKAFF	0.194	0.575	3
VJ-4007.53.2	CASGNEQFF	0.144	0.636	4
