# Supplementary material for: Reconstitution of EBV-directed T cell immunity by adoptive transfer of peptide-stimulated T cells in a patient after allogeneic stem cell transplantation for AITL
Source: PLoS Pathog. 2022 Apr 22;18(4):e1010206. doi: 10.1371/journal.ppat.1010206 (PMC9067708; doi:10.1371/journal.ppat.1010206)
Supplement: S5 Table — (PDF) [file ppat.1010206.s014.pdf]

EPL-specific T cells			
**sample**	**No. TCR clonotypes**	**proportion of $CD8^+$ TCR clonotypes (%)**	**frequency in $CD8^+$ T cells (%)**
**donor PBMC**	32	1.64	3.16
**T cell product**	40	8.49	15.07
**patient, day 60**	2	1.85	3.67
**patient, day 120**	34	5.27	15.43
**patient, day 180**	33	6.06	11.05
**patient, day 232**	34	4.60	7.09

RAK-specific T cells			
**sample**	**No. TCR clonotypes**	**proportion of $CD8^+$ TCR clonotypes (%)**	**frequency in $CD8^+$ T cells (%)**
**donor PBMC**	17	0.87	1.06
**T cell product**	28	5.94	30.26
**patient, day 60**	4	3.70	4.78
**patient, day 120**	21	3.26	5.06
**patient, day 180**	21	3.85	3.92
**patient, day 232**	18	2.44	1.21

HPV-specific T cells			
**sample**	**No. TCR clonotypes**	**proportion of $CD8^+$ TCR clonotypes (%)**	**frequency in $CD8^+$ T cells (%)**
**donor PBMC**	6	0.31	1.21
**T cell product**	9	1.91	29.47
**patient, day 60**	0	0.00	0.00
**patient, day 120**	6	0.93	2.37
**patient, day 180**	5	0.92	2.45
**patient, day 232**	3	0.41	0.19
