# Supplementary material for: Reconstitution of EBV-directed T cell immunity by adoptive transfer of peptide-stimulated T cells in a patient after allogeneic stem cell transplantation for AITL
Source: PLoS Pathog. 2022 Apr 22;18(4):e1010206. doi: 10.1371/journal.ppat.1010206 (PMC9067708; doi:10.1371/journal.ppat.1010206)
Supplement: Attachment — Submitted filename: Response_to_Reviewers.pdf [file ppat.1010206.s015.pdf]

**PPATHOGENS-D-21-02534**

Dear Editor,

Thank you for considering our manuscript "Reconstitution of T cell immunity against EBV in the immunocompromised host by adoptive transfer of peptide-stimulated T cells after allogeneic stem cell transplantation" (PPATHOGENS-D-21-02534).

Please find enclosed our response to the reviewers' comments. Changes to the manuscript are indicated by line number. We also include two versions of the revised manuscript: one with highlighted changes and a clean one.

The revised manuscript contains modifications of Figures 2C and 3A, as well as 6 new supplementary figures in line with the reviewers' suggestions. The text has been altered at several points and the discussion has been substantially modified to address the reviewers' requests.

We thank the reviewers for their profound, constructive, and very helpful comments, as they have led to a substantial improvement in the article in terms of clarity, discussion, and result presentation. We hope the manuscript in its current version is now suitable for publication.

Thank you very much in advance,

Armin Gerbitz

References to changes in the manuscript are indicated by line number and appear highlighted in the manuscript.

## Reviewer 1

**Part I - Summary**

**"Lammoglia Cobo et al. describe a case of AITL relapse soon after allogeneic hematopoietic cell transplantation treated with DLI, rituximab and *ex vivo* expanded EBV antigen-specific T cells. A particular focus was put on the characterization of the TCR clonotype content of the product and the monitoring of likely EBV-specific T cells post-transfer.**

**Such a strategy has been described several times, including the characterization of the product and immunomonitoring using TCR sequencing. However, the manufacturing method includes novel features (polyspecific antigen-specific CD8 T cell expansion guided by HLA allotypes) and the focus of the authors on the clonotypic characterization of the product and immune reconstitution offer interesting insights. Nonetheless, the authors should consider the following aspects to make their findings more impactful and their report more complete."**

We thank the reviewer for his insights and have therefore emphasized both multispecific antigen-driven expansion of T cells and the molecular characterization of both the cellular product and *in vivo* reconstitution in the Abstract (lines 30-33, 36-37), Introduction (lines 90-95), and Discussion (lines 330-337).

**Part II – Major Issues**

**"1- The expanded T cells were directed against antigens from BZLF1, EBNA3A and EBNA1. *In vivo*, BZLF1-specific T cells expanded more that the EBNA-1 specific T cells (which was not the case *ex vivo*). Did the AITL at relapse (or at diagnosis) express the targeted proteins? Not all AITL express latency type 3 proteins and, as such, the AITL expression of BZLF1 should be confirmed (especially in a context in which AITL relapse clearly appears to be the cause of the rising EBV viral load)? Otherwise, what can account for this difference (exhaustion markers expression, poor functional features, immunodominance, etc)? Additional characterization of the product along these lines would improve the manuscript."**

To verify EBV expression of AITL, we used EBER *in situ* hybridization (IsH) of the lymph node at diagnosis. Only very rarely EBER$^+$ cells were seen (new S2A Fig). AITL is often accompanied by activated EBV$^+$ B cells in the tumor microenvironment; however, we did not observe it in this case (new S2B Fig.). Unfortunately, no lymph node biopsies from relapse were available for further analysis, as this relapse appeared to be leukemic.

Adoptive transfer of EBV-specific T cells was given as a curative treatment specifically for high EBV viremia. The leukemic relapse after transplant was initially treated with a conventional DLI; however, the EBV load was high and associated with severe symptoms. In this study, we decided to focus on the expansion and *in vivo* survival of EBV-specific T cells to treat EBV reactivation post-transplant. The fact that the AITL was EBV$^-$ indicates there was a separate infection site which produced the EBV viremia. However, simultaneous AITL relapse and increased EBV viral titers in blood suggest a strong association between AITL and EBV infection.

We feel that potential mechanisms to explain differences in expansion between BZLF1- and ENBA1-specific T cells are their differentiation stages and the availability of their target epitope. Although cells for the 3 specificities mostly lack CCR7 expression (CCR7$^-$CD45RA$^-$), both lytic BZLF-1 EPL- and RAK-specific T cells had a stronger CD62L expression than latent EBNA1 HPV-specific T cells (new S5 Fig). This difference and CD62L association with central memory may indicate a higher proliferative strength, a stronger expansion (as seen in Fig 3C), less exhaustion, and better homing ability of the lytic-specific compartment. Otherwise, an alternative hypothesis to the expansion difference between lytic and latent-specific cells is availability of target

epitope: The strong expansion of EPL- and RAK-specific T cells in peripheral blood and cytokine release (new S4 Fig) happened directly after transfer, when ongoing EBV viremia may have provided enough lytic epitope presentation. EBV viremia decrease over time and lesser epitope availability would explain for few clonotypes to remain and survive long-term. On the other hand, HPV-specific T cells strongly expanded *ex vivo* but not as strongly in peripheral blood *in vivo*, either due to its homing to an active infection site, such as a lymph node, or the unavailability of its target antigen EBNA1.

We have included these points in the Results (lines 98-100 and 147-150), Discussion (lines 338-344 and 357-376), Material and Methods (lines 455-456) and as three new supplementary figures (S2, S4, and S5 Fig).

**"2- The clonotype composition of sorted cells did show significant overlap between Ag-specific populations. Although resolved by the application of "filters," doubts remain regarding the true Ag-specificity of the sorted cells and whether sorting was stringent enough. Functional (cytokine secretion, degranulation) tests using sorted cells in the presence of their target peptides vs non-target peptides would convince further."**

Our wording in the manuscript text may have suggested that the overlap between the three sorted populations was larger than it actually was; however, these overlapping clonotypes had only minor frequencies, compared to very dominant clonotypes per specificity. As an example, HPV-specific TCR VJ-4001.53.1 had a 38.8% frequency in HPV-sorted cells, compared to 0.1% in EPL and 0.2% in RAK. For this reason, we believe that the composition of each epitope-specific compartment was dominated by higher-frequency clonotypes, while we can always expect to find cells with very low frequencies as contaminants.

To confirm this point, we have included in the revised manuscript:

- Multimer sorting purity: Gating for multimer sorting was stringent enough to achieve an efficiency above 98% for all epitopes (new S6 Fig).
- Removal of contaminant cells through stringent frequency cut-offs and multimer ternary exclusion (S7 Fig)

To highlight these points (sort stringency and contaminant removal) and avoid confusion, we have created a new S6 Fig and modified Results (lines 162-163, 182, 203-206, and 209-212), Discussion (line 401), and Fig 2C left panel.

We used multimer sorting in the article as party of quality control to analyze the epitope-specific compartment in greater detail. This was not used as part of the manufacturing protocol, as multimer binding might alter T cell functionality and lead to artificial activation. For this reason, the functional status of the sorted populations wasn't explored. Instead, we showed functionality of the T cell product by restimulation with EPL, RAK, and HPV peptides, which lead to IFN-$\gamma$ production as an equivalent (Fig 1C).

**Part III – Minor Issues**

**"1- The description of the case. The report is at times confusing – to help with the flow of the manuscript, the content of Table S1 should be described at the beginning and a timeline provided (time from chemotherapy to transplant unclear, various treatments, etc) in the body of the manuscript."**

We thank the reviewer for this suggestion: We have rearranged the manuscript to start with the description of the case in Results (lines 98-109), a new figure with histological information (S2 Fig), and a timeline (S3 Fig) as a supplement to S1 Table. We cannot show particular dates of chemotherapy due to privacy regulations; however, we have assigned the day of allogeneic transplantation as day 0 and calculated chemotherapy dates accordingly.

**“2- The occurrence of severe GVHD after adoptive transfer should be discussed (role of DLI, ATCT?, impact of steroid-therapy on reconstitution and in this case, the occurrence of a fatal infectious outcome). Likewise, DLI and Rituximab seem to have had a greater therapeutic impact than ATCT (not clear that it was administered in the context of a rising EBV viral load). The potential implications of that should be discussed as well.”**

Thank you for having raised this issue. GvHD is an important, yet slightly confusing point, which we deliberately left out in the initial manuscript. Given that both reviewers asked for this information, we have added a section in the Discussion (lines 378-389) and a new figure (S9 Fig) and hope this clarifies the issue.

We expected a decrease in EBV viremia after DLI and Rituximab infusion; however, EBV viremia kept increasing until it reached its peak simultaneously with the last dose of Rituximab on day 89 and EBV-related symptoms remained. While EBV titers started declining after this point, we saw an expansion of the EBV-specific T cells and cytokine release directly after adoptive transfer. This expansion points to the availability of their target antigens still being present. We altered Fig.3A to reflect more precisely the days of Rituximab infusion and have included these points in the Discussion (lines 352-356).

**“3- Fig 4 shows that the donor PBMCs contain several of the abundant product clonotypes. Although the author provides a rationale to link ATCT to EBV-specific immune reconstitution, they do not rule out the possibility that clonotypes in the DLI contributed to the immune reconstitution. This should be mentioned and discussed.”**

Thank you for raising this important point. DLI may indeed have contributed to the surviving EBV memory T cell pool and we cannot formally exclude this. However, we were able to track EBV-specific T cell clonotypes coming from the adoptive transfer that persisted long-term in the patient.

We included these points in the Discussion (lines 352-356).

**“4- The EBV viral load measurement method is not specified – plasma/serum or whole blood? Commercial assay or LDT?”**

We have included this information in the Materials and Methods section (lines 450-454).

**“5- As opposed to what is described in the text, Fig S1 does not show data on IFNg.”**

We apologize for this error. We have now altered the figure to show cytokine secretion as S3 Fig and re-arranged the other supplementary figures accordingly.

## Reviewer 2

**Part I – Summary**

**"The authors demonstrate that Epstein Barr virus (EBV) derived peptide stimulation of peripheral blood mononuclear cells (PBMCs) derived from a patient with recurrent angioimmunoblastic T cell lymphoma (AITL) after transplantation leads to the expansion of EBV specific CD8$^+$ T cells. These are primarily directed against peptides from BZLF1 and EBNA1. Particularly EBNA1 specific CD8$^+$ T cells were dominated by one TCR clonotype. Several of the identified EBV specific TCR clonotypes expand after transfer into the patient and are maintained for at least 8 months. From these data the authors conclude that a diverse set of EBV specific clonotypes can be expanded with their protocol and transferred for treatment of AITL, reestablishing EBV specific immune control after stem cell transplantation.**

**Even so the authors apply TCR clonotype tracing to follow adoptively transferred EBV specific T cells in one bone marrow transplant patient for the first time, it is unclear what new insights they gain from these studies. Persistence of such adoptively transferred T cells has previously been documented for up to 18 months (Heslop et al., Nat Med 1996). Therefore, the authors should address in more detail how the adoptively transferred T cell products control the pathogenic T cell expansion in this patient and if there are differences between the detected EBV specific TCR clonotypes."**

We thank the reviewer for pointing this out. The novel aspects of this article are the molecular characterization at a clonotype level of adoptively-transferred EBV-specific T cells against selected epitopes of known HLA-restriction Therefore, we have emphasized these aspects of this article in the Abstract (lines 30-33, 36-37), Introduction (lines 90-95), and Discussion (lines 330-337).

The adoptive transfer of EBV-specific T cells was decided as a curative treatment for high EBV viremia and was not aimed to control AITL. However, simultaneous AITL relapse and increased EBV viral titers in blood suggest a strong association between AITL and EBV, as mentioned in the literature.

We feel potential mechanisms to explain differences in expansion between BZLF1- and EBNA1-specific T cells are their differentiation stages and availability of their target epitope. Although cells for the 3 specificities mostly lack CCR7 expression (CCR7$^-$CD45RA$^-$), both lytic BZLF-1 EPL- and RAK-specific T cells had a stronger CD62L expression than latent EBNA1 HPV-specific T cells (new S5 Fig). This difference and CD62L association with central memory may indicate a higher proliferative strength, a stronger expansion (as seen in Fig 3C), less exhaustion, and better homing ability of the lytic-specific compartment. Otherwise, an alternative hypothesis to the expansion difference between lytic and latent-specific cells is availability of target epitope: The strong expansion of EPL- and RAK-specific T cells in peripheral blood and cytokine release (new S4 Fig) happened directly after transfer, when ongoing EBV viremia may have provided enough lytic epitope presentation. EBV viremia decrease over time and lesser epitope availability would explain for few clonotypes to remain and survive long-term. On the other hand, HPV-specific T cells strongly expanded *ex vivo* but not as strongly in peripheral blood *in vivo*, either due to its homing to an active infection site, such as a lymph node, or the unavailability of its target antigen EBNA1.

We have emphasized the difference between T cell clonotypes in the Results (lines 147-150), Discussion (lines 357-376), and as two new supplementary figures (S4-S5 Fig).

**Part II – Major Issues**

**"1. The composition of the uncontrolled EBV infection in the investigated patient should be characterized in more detail. Was EBV detected in the patient's AITL cells? Were there also elevated EBV titers in the CD4$^{+}$ T cell negative fraction?"**

To verify EBV expression of AITL, we used EBER *in situ* hybridization (IsH) of the lymph node at diagnosis. Only very rarely EBER$^{+}$ cells were seen (new S2A Fig). AITL is often accompanied by activated EBV$^{+}$ B cells in the tumor microenvironment; however, we did not observe it in this case (new S2B Fig.).

Adoptive transfer of EBV-specific T cells was applied as a curative treatment specifically for high EBV viremia. In this study, we decided to focus on the expansion and *in vivo* survival of EBV-specific T cells to treat EBV reactivation post-transplant. The fact that the AITL was EBV$^{-}$ indicates there was a separate infection site which produced the EBV viremia. However, simultaneous AITL relapse and increased EBV viral titers in blood suggest a strong association between AITL and EBV.

We have included this topic in the Results (lines 98-100), Discussion (lines 338-344), Material and Methods (lines 455-456), and a new supplementary figure (S2 Fig).

**"2. The authors report the contribution of several TCR clonotypes to the T cell response against BZLF1 and EBNA1 specific T cell reactivities. What is the difference between the detected TCR clonotypes? Do the authors have any evidence that some of these were of higher affinity for the identified epitopes? Did this correlate with their expansion in vitro or in vivo?"**

In the response to "Part I- Summary", we addressed the difference in expansion between the BZLF-1- and EBNA1-specific T cells in terms of differentiation phase and target antigen availability. Here, we'd like to address the question of affinity.

In healthy individuals, EBV epitope-specific T cells tend to have a wide range of affinities (Trautmann *et al.* 2022, Zhang *et al.* 2016), with outliers removed during T cell development. T cells with strong binding to self-peptide MHC molecules are modified through immune tolerance mechanisms; on the other hand, those unable to bind disappear to the repertoire (Xing *et al.* 2012). Use of multimer binding has a bias for TCRs with higher affinities because the affinity threshold required for multimer binding is higher than the one for T cell activation (Laugel *et al.* 2007). Therefore, we would conclude both BZLF1- and EBNA1-specific clonotypes identified in this study to have relatively higher affinity levels.

As manufacturing depends on the donor's EBV-specific T cell compartment, we do not believe peptide stimulation produced significant changes on TCR clonotype dominance within epitope-specific populations, nor that the hierarchies depend on higher or lower affinities of T cell clonotypes. Table 2 shows the most dominant T cells per epitope-specificity were already dominant in the cellular product and even before peptide stimulation.

We included this information in the Discussion (lines 402-405).

**"3. Did certain TCR clonotypes recognize the autologous AITL cells more efficiently than others? Was there any difference in EBNA1 versus BZLF1 specific T cell recognition of autologous AITL, if this actually harbors EBV?"**

As discussed in the first question of "Part II- Major Issues", AITL cells were EBV$^{-}$. For this reason, EBV-specific T cells are not expected to recognize AITL cells. However, our goal was to target rising numbers of EBV titer in blood. We observed cytokine release and EBV-specific T cell clonotype expansion after adoptive transfer in the context of EBV viremia, which points to recognition of their target antigen *in vivo*. Furthermore, some of these clonotypes survive long-term and contribute to EBV monitoring.

**Part III – Minor Issues**

**"1. The title should indicate that the study reports only one patient and that this patient suffered from AITL."**

As suggested, we have modified the title to include these two points.
